# Synthesis, DNA-Binding and Antiproliferative Properties of Acridine and 5-Methylacridine Derivatives

**DOI:** 10.3390/molecules17067067

**Published:** 2012-06-08

**Authors:** Rubén Ferreira, Anna Aviñó, Stefania Mazzini, Ramon Eritja

**Affiliations:** 1 Institute for Research in Biomedicine (IRB Barcelona), Baldiri Reixac 10, E-08028 Barcelona, Spain; Email: ruben.ferreira@irbbarcelona.org (R.F.); anna.avinyo@irbbarcelona.org (A.A.); 2 Institute for Advanced Chemistry of Catalonia (IQAC), CSIC, CIBER-BBN Networking Centre on Bioengineering, Biomaterials and Nanomedicine, Jordi Girona 18, E-08034 Barcelona, Spain; 3 Department of Agro-Food Molecular Sciences (DISMA), University of Milan, Via Celoria 2, 20133 Milan, Italy; Email: stefania.mazzini@unimi.it

**Keywords:** acridine, DNA-binding drugs, solid-phase synthesis, G-quadruplex, NMR

## Abstract

Several acridine derivatives were synthesized and their anti-proliferative activity was determined. The most active molecules were derivatives of 5-methylacridine-4-carboxylic acid. The DNA binding properties of the synthesized acridines were analyzed by competitive dialysis and compared with the anti-proliferative activities. While inactive acridine derivatives showed high selectivity for G-quadruplex structures, the most active 5-methylacridine-4-carboxamide derivatives had high affinity for DNA but showed poor specificity. An NMR titration study was performed with the most active 5-methylacridine-4-carboxamide, confirming the high affinity of this compound for both duplex and quadruplex DNAs.

## 1. Introduction

DNA-intercalating drugs are planar molecules composed by several fused aromatic rings that form stacks between DNA base pairs, thus reducing the opening and unwinding of the double helix. Each intercalating drug binds strongly to particular base pairs as a result of several interactions, ranging from van der Waals forces to the formation of hydrogen bonds with adjacent nucleobases [1,2].

Telomeres are specialized DNA-protein structures at the termini of chromosomes crucial for chromosomal stability and accurate replication. Human telomeric DNA contains tandem repeats of the sequence TTAGGG. The guanine-rich strand can fold into four-stranded G-quadruplex structures involving G-tetrads, which are currently an attractive target for the development of anti-cancer drugs [3,4]. Acridine derivatives inhibit telomerase, presumably through their interaction with the G-quadruplex structures found in telomeric DNA [5,6]. A wide range of small molecules have been studied as quadruplex-binding and stabilizing ligands [7]. Most of these share common structural features, namely: (i) a planar heteroaromatic chromophore, which stacks by π-π interactions onto the G-quartet motif at the terminus of a quadruplex; and (ii) short alkyl chain substituents usually terminated by an amino group that is fully cationic at physiological pH. The precise nature of these substituents has been found to influence quadruplex affinity and selectivity [8,9].

Topoisomerase alters DNA topology through the decatenation and relaxation of supercoiled DNA [10]. By unwinding double-stranded DNA, this essential enzyme allows for normal cellular functions, such as replication and transcription [10]. DNA topoisomerases exist in various eukaryotic and prokaryotic forms [11] and are classified in two large groups named type I and type II. Topoisomerase-targeting anti-cancer drugs can be divided into two broad classes depending on their mechanism of action, either catalytic inhibitors or “topoisomerase poisons” [12]. The latter can be further subclassified into two groups: non-intercalating compounds, such as etoposide, and intercalators, like amsacrine and doxorubicin [13]. Intercalators act by forming ternary complexes with topoisomerases and DNA to inhibit re-ligation. However, the selectivity of intercalators for a particular DNA sequence is very low. Most often, selectivity is obtained from interactions of side-chain substitution in the major and minor grooves [14]. Another strategy to improve the selectivity of intercalating drugs is by linking several intercalating units. Various authors have described the synthesis of bis- or tris-intercalating drugs that show promising activity and selectivity [15,16,17,18].

The consensus is that acridine analogs target DNA through intercalation and disrupt enzyme recognition and/or association [19]. Acridine-4-carboxamides are a series of DNA intercalating topoisomerase poisons that show anti-tumor activity [20]. Among these, *N*-[2-(dimethylamino)ethyl]acridine-4-carboxamide (DACA) is a DNA-intercalating agent that inhibits both topoisomerase I and II [21] and is currently in phase II clinical trials. There are tight correlations between ligand structure, cytotoxicity and DNA-binding kinetics [22].

In the present study, we designed, synthesized and studied acridine and 5-methylacridine derivatives as potential anti-tumoral agents. During the selection of the acridine derivatives, we considered solid-phase methods for the preparation of the target compounds. Recently, we used peptide [23] and oligonucleotide chemistry [24] to prepare DNA-intercalating oligomers with several backbones for the assembly of a number of intercalating units. The modular character of solid-phase methods allows the rapid preparation of larger molecules that have G-quadruplex specific affinity [25,26]. The cytotoxicity of the acridine and 5-methylacridine derivatives to a tumoral cell line was assessed in MTT cell viability assays, thus identifying compounds exerting anti-tumoral activity. The DNA binding properties of the synthesized acridines were studied by competitive dialysis experiments. The affinity of the most active 5-methylacridine-4-carboxamide derivative to G-quadruplex telomeric and duplex DNA sequences was further analyzed by NMR. This analysis confirmed the binding of this compound to both quadruplex and duplex DNA sequences.

## 2. Results and Discussion

### 2.1. Synthesis of the Acridine Derivatives

**Figure 1:**
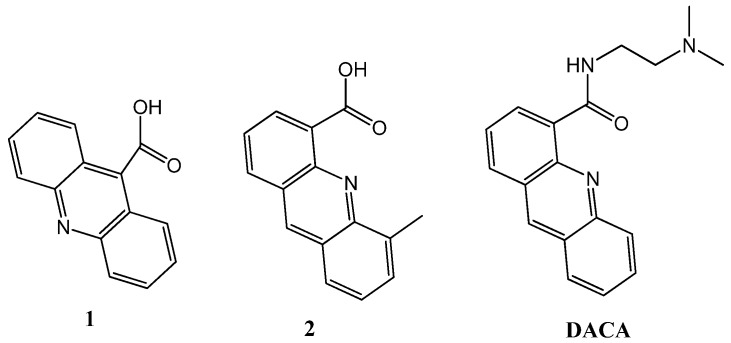
Chemical structures of *N*-[2-(dimethylamino)ethyl]acridine-4-carboxamide (DACA) and starting compounds **1** and **2**.

**Figure 2:**
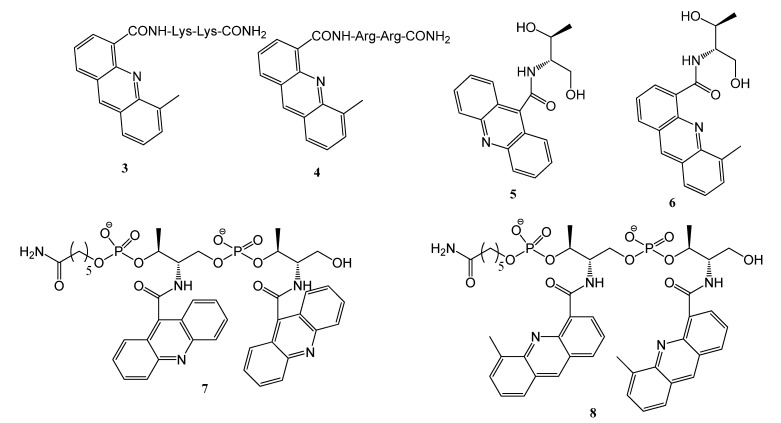
The acridine and 5-methylacridine derivatives synthesized in this study.

We selected acridine-9-carboxylic acid (**1**) and 5-methylacridine-4-carboxylic acid (**2**) as starting compounds for the preparation of the new derivatives (Figure 1). Compound **1** is commercially available and has no anti-proliferative properties [24]. Compound **2** has been described as an intermediate in the synthesis of the bis-acridine derivatives of DACA [18]. Thus, in this study, we undertook the synthesis of compounds with this unit. Two types of derivatives were prepared. First, the replacement of the dimethylamino group of DACA for two residues of lysine (**3**) or arginine (**4**) was studied (Figure 2). These derivatives were prepared to check whether the protonable dimethylamino group can be replaced by amino acids with amino (Lys) or guanidino (Arg) groups. The synthesis of compounds **3** and **4** was performed by a standard solid-phase peptide approach using Fmoc-amino acids. After assembly of the dipeptide carboxylic acid **2** was coupled to the α-amino group of the dipeptides. Next we studied the possibility to generate compounds holding two units of the 5-methylacridine ring present in the 5-methyl derivative of DACA [18]. To join the units, we chose the L-threoninol backbone connected by phosphodiester links [27] for several reasons. The length of the threoninol linker is compatible with DNA structure and can be obtained in an enantiomerically pure form.

**Scheme 1:**
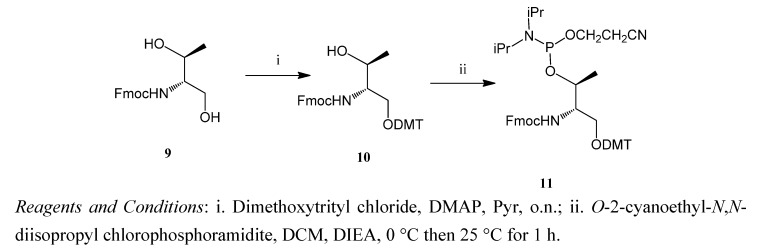
Synthesis of the threoninol phosphoramidite derivative.

Threoninol has two distinct hydroxyl groups and one amino group. The intercalating agent can be attached at the amino group position, thus leaving the hydroxyl groups to build the backbone using standard solid-phase oligonucleotide methods [23,27]. To this end, the primary hydroxyl group of threoninol was protected by the 4,4′-*O*-dimethoxytrityl (DMT) group and the secondary alcohol was used to prepare the phosphoramidite derivative (Scheme 1).

**Scheme 2:**
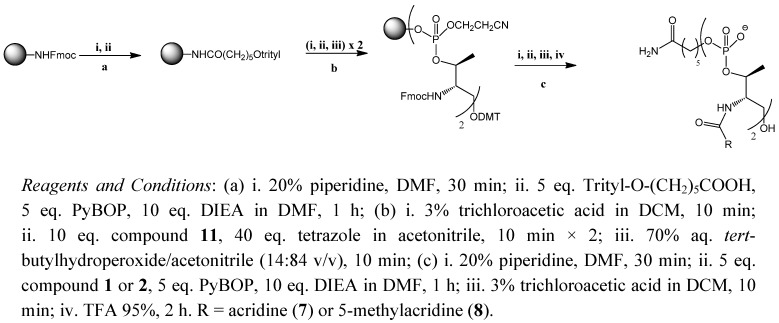
Solid-phase synthesis of acridine oligomers.

For the synthesis of intercalating oligomers **7** and **8**, the threoninol backbone was grown on solid-phase, and then the intercalating agent was assembled on solid support. This strategy is more convenient for rapid synthesis, as it is unnecessary to construct each monomer with its intercalating agent, as described previously [23]. Here we report a hybrid synthesis. First, the phosphoramidite described above was assembled into a dimer (Scheme 2).

Modified standard phosphoramidite chemistry was used. This consists of cycles of 3 chemical reactions: (1) removal of the DMT group with 3% trichloroacetic acid in dichloromethane; (2) phosphoramidite coupling using 10-fold excess of phosphoramidite and 40-fold excess of tetrazole and (3) oxidation of phosphite to phosphate with hydroperoxide solution in acetonitrile. The use of iodine for the oxidation of phosphites was avoided as we have previously observed some side products attributable to the premature removal of the Fmoc group. Also a capping reaction with acetic anhydride and *N*-methylimidazole was omitted in order to avoid the acetylation of the amino group observed in the synthesis of oligonucleotide-peptide conjugates using Fmoc-amino acids [28]. To guarantee a high coupling yield, two consecutive phosphoramidite coupling reactions were systematically performed. Finally, Fmoc groups of threoninol were removed to allow coupling of carboxylic acids **1** or **2**, thereby providing the desired dimers **7** and **8** in satisfactory yields. Monomeric threoninol derivatives **5** and **6** were prepared as described [23].

### 2.2. Cell Viability Assay

The *in vitro* cytotoxicity of the compounds **1**–**8** was evaluated by colorimetric assays with a tetrazole salt (MTT) on the human colon carcinoma HBT38 cells. This assay is based on the capacity of living cells to incorporate and reduce the tetrazole salt. This reaction can be followed by the absorbance change of the reduced and oxidized forms. The reduction is observed only in living cells and the color intensity is directly correlated with the number of viable cells. The IC_50_ values for compounds **1**–**8** are given in Table 1.

**Table 1:** Anti-proliferative activity: n.a. not active.

Compound	IC_50_ (µM) HBT38
**1**	n.a.
**2**	75
**3**	n.a.
**4**	n.a.
**5**	n.a.
**6**	60
**7**	n.a.
**8**	25

The acridine derivatives **1**, **5** and **7** did not show anti-tumoral activity. These results were expected as acridine-9-carboxylic acid [24] as well as 2-aminoethylglycine and aminoprolyl oligomers have no antiproliferative activity [24,25]. On the other hand, the 5-methylacridine-4-carboxylic acid derivatives **2**, **6** and **8** had moderate activity, dimer **8** being the most active compound. In contrast, the peptide derivatives **3** and **4** lost this activity. This is partially in agreement with the antiproliferative properties assigned to 5-methylacridine-4-carboxylic acid derivatives [17,18,20,21,22]. We expected some activity for peptide derivatives **3** and **4** because these compounds have a positively charged residue linked to the carboxylic acid function as in the case of 5-methyl-*N*-[2-(dimethylamino)ethyl]acridine-4-carboxamide that have potent antitumor activity [21]. But in our study the addition of lysine and arginine residues instead of the *N*-dimethylaminoethyl group were detrimental for the antiproliferative activity. Also, literature reports that 5-substituted bis(acridine-4-carboxamide) derivatives have good anticancer activities [18]. In this work we observed the best antiproliferative activity for the dimer carrying two 5-methylacridine-4-carboxamide units linked through threoninol phosphate backbone confirming the increase of activity by linking two DNA intercalating units. To our knowledge this is the first bisintercalating molecule linked by phosphate bonds with increased activity. In order to confirm that the activity may be mediated by DNA binding, competitive dialysis experiments were performed. 

### 2.3. Competitive Dialysis Studies

**Figure 3:**
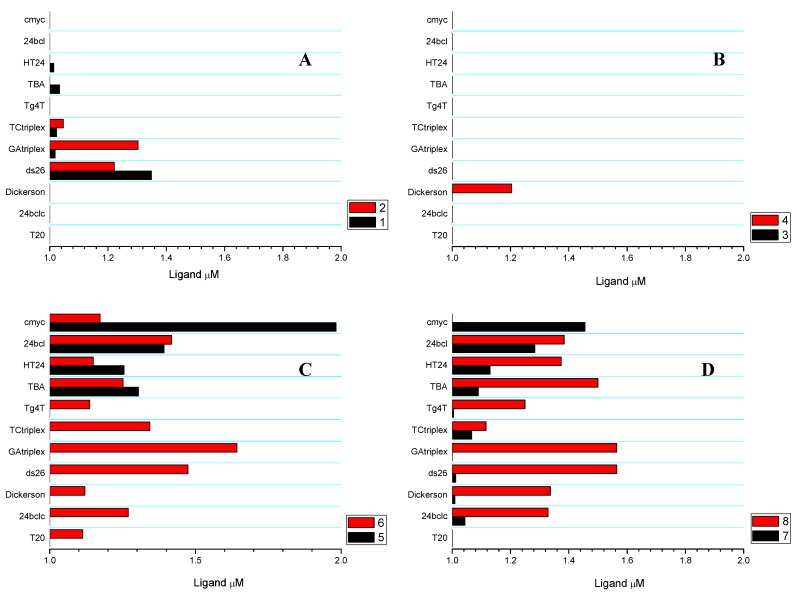
Competitive dialysis assay: The amount of ligand bound to each DNA structure is shown as a bar graph.

To evaluate the selectivity and the affinity of intercalating derivates for DNA structures, we performed a competitive dialysis experiment using 11 nucleic acid structures [25]. The DNA sequences were selected to represent all the potential DNA structures that may be present at physiological pH: single-stranded, duplex, parallel and antiparallel triplexes and quadruplexes. As models for single-stranded structures, we used T20 and 24bclc. As duplexes, we used a self-complementary sequences Dickerson–Drew dodecamer (Dickerson) and a 26 mer (ds26). A parallel triplex (TC triplex) and an antiparallel triplex (GA triplex) were also prepared by mixing a hairpin Watson–Crick sequence and the corresponding triplex-forming sequence. Finally, the following G-quadruplex sequences were prepared: the tetramolecular parallel G-quadruplex TG_4_T [29]; the antiparallel thrombin-binding aptamer (TBA) [30]; the human telomere sequence (HT24) [31]; and the promoter sequences of *c-myc* (cmyc) [32] and *bcl-2* (24bcl) [33]. The amount of bound ligand was directly proportional to the binding constant for each DNA structure [34,35]. Figure 3 shows the oligonucleotide affinity for each compound and Figure 3A the results for acridine and 5-methyl acridine carboxylic acids **1** and **2**. Acridine-9-carboxylic acid **1** interacts only with duplex ds26, the methyl derivative **2** induced a change in the affinity showing also a triplex preference. While acridine derivatives **5** and **7** showed quadruplex selectivity, the methyl analogs **6** and **8** lost most of the selectivity, although they presented a higher affinity for most DNA sequences (Figure 3C and Figure 3D). In contrast, the peptide derivatives **3** and **4** presented lower binding affinity for DNA (Figure 3B). Comparing the competitive dialysis results with the antiproliferative activity we can observe that the peptide derivatives **3** and **4** have both low affinity for any DNA molecule and no antiproliferative activity. Acridine derivatives **5** and **7** have interesting binding affinities for DNA quadruplex sequences but this affinity does not trigger inhibition of cell grown of HBT38 cancer cell lines. This is agreement with previous work on 2-aminoethylglycine and aminoprolyl acridine oligomers [24,25]. Finally, the most antiproliferative compounds **6** and **8** have a strong affinity for all type of DNA sequences. Although we cannot discard the hypothesis that the antiproliferative activity of these compounds is due to direct binding to proteins, we can hypothesize that the antiproliferative activity of these compounds may be mediated by DNA binding. This is in agreement with the inhibition of DNA topoisomerases described for this family of compounds [12,17,18,20,21,22].

### 2.4. NMR Spectroscopy

On the basis of cell viability studies the compound **8** demonstrated the highest anti-tumoral activity. Competitive dialysis experiments also show that compound **8** has affinity to all types of DNA sequences in spite of the presence of a negative phosphate backbone that may hinder binding with the DNA polyanionic molecule. As it is the first time that a bisintercalating molecule having a negatively charged phosphate backbone is shown to have DNA binding properties we confirmed that compound **8** is able to bind to both duplex and quadruplex DNA molecules. First we tried the classical methods such UV- and fluorescence-based melting assays [36], fluorescence intercalator displacement assay [37], and mass spectrometry [38]. But any of these methods provide conclusive results and some of these methods were not compatible with the fluorescence properties of the acridine derivatives. For these reasons and in order to confirm the interaction with DNA, **8** was titrated into a solution of the quadruplex model (T_2_AG_3_)_4_ of the human telomere sequence and the resulting mixtures were analyzed by ^1^H-NMR. Similar experiments were performed with the starting carboxylic acid **2**. T_2_AG_3_ is a short model sequence contained in the HT24 oligonucleotide. This oligonucleotide has been used previously for NMR characterization of drug binding on telomeric DNA sequences [39]. The short oligonucleotide has a more simple NMR spectrum than HT24 sequence, thus facilitating the study of the interactions of the drug with the DNA sequence.

**Figure 4:**
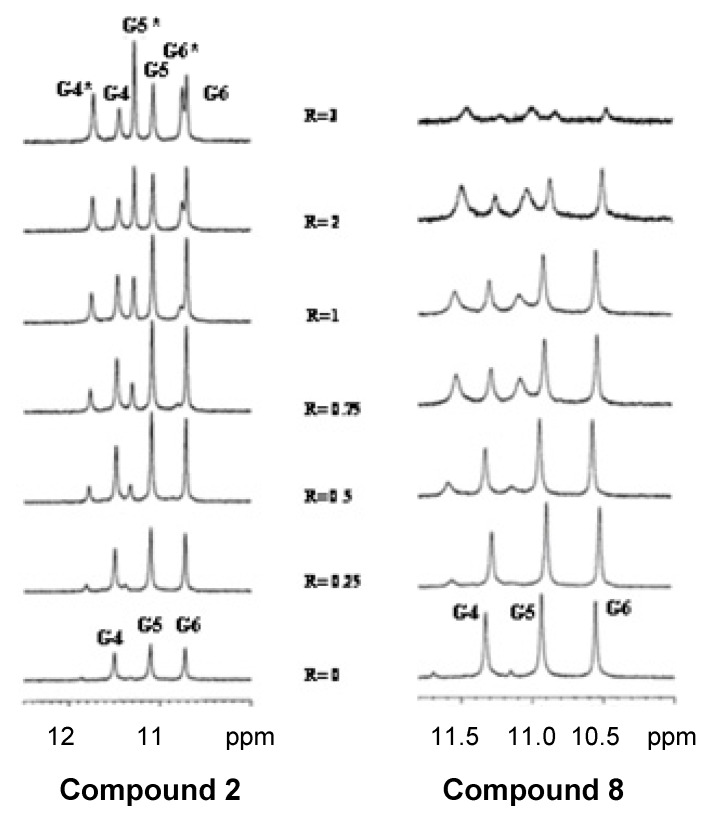
Imino proton region of (TTAGGG)_4_ resulting from the titration of the quadruplex with compounds **2** and **8**.

The NMR spectra for the quadruplex showed three signals in the region of 10–12 ppm, belonging to Hoogsteen-bound guanine imino proton of the G quartets (Figure 4). During the addition of **8** to the quadruplex solution until reaching a ratio R = [**8**]/[(T_2_AG_3_)_4_] = 3, the imino proton signals caused by G4, G5 and G6 splits in the downfield-shifted region were related to the bound quadruplex form. This result confirms an **8** quadruplex interaction complex associated with drug toxicity. The imino protons signals were broad and disappeared at high R indicating that more than one species in equilibrium were present in solution (Figure 4).

**Figure 5:**
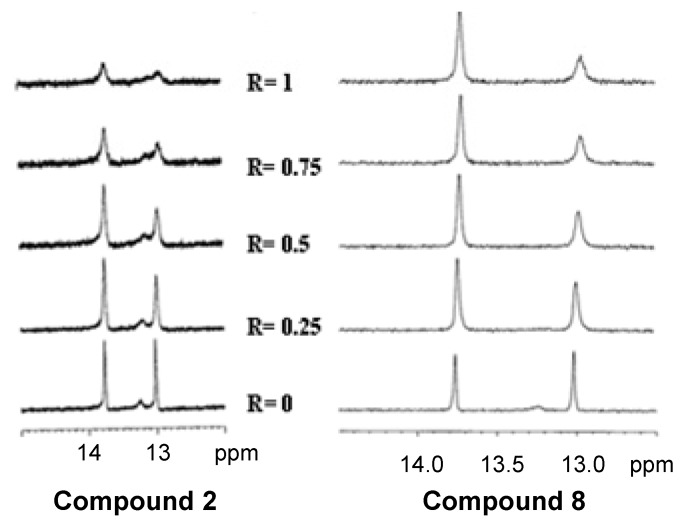
Imino proton region of (CGATCG)_2_ resulting from the titration of the duplex with compounds **2** and **8**.

The oligonucleotide sequence 5′-CGATCG-3′ was used as model for a DNA duplex. In the region of 13–14 ppm, the NMR spectra of the duplex showed two signals, these belonging to imino proton of A3T4 and G2C5 (Figure 5). 

During the addition of **8** to the duplex solution until reaching the ratio R = [**8**]/[(CGATCG)_2_] = 1, the imino proton signals caused by G2 decreased the intensity of the signal, thereby indicating a preferred site of interaction at this level. The equivalent experiments with compound **2** showed slight differences. During the addition of **2** to the quadruplex solution until reaching the ratio R = [**2**]/[(T_2_AG_3_)_4_] = 3, the imino proton signals caused by G4, G5 and G6 split in the downfield-shifted region, thus indicating a binding interaction. In this case the imino proton signals were not broad at high R (Figure 4), indicating that only one bound species was present in the solution. Free quadruplex species structure was still present at this ratio.

During the addition of **2** to the duplex solution until reaching the ratio R = [**2**]/[(CGATCG)_2_] = 1, the imino proton signal decreased in the intensity and was broad, suggesting the presence of multiple binding sites and complex equilibria (Figure 5).

In summary, NMR titration experiments confirm the interaction of compound **8** with both duplex and quadruplex DNA sequences, even when the quadruplex complex equilibria in solution were involved, whereas the interaction was more specific with the double helix, compound **2** showing the opposite behavior.

## 3. Experimental

### 3.1. Oligonucleotides and General Information

Standard phosphoroamidites and reagents for DNA synthesis were purchased from Applied Biosystems and from Link Technologies. The synthesis of the oligonucleotides was performed at a scale of 1 μmol on an Applied Biosystem DNA/RNA 3400 synthesizer by solid-phase 2-cyanoethylphosphoroamidite chemistry. The following sequences were prepared: T20: d(5′-TTT TTT TTT TTT TTT TTT TT-3′), 24bclc: d(5′-CCC GCC CCC TTC CTC CCG CGC CCG-3′), Dickerson: d(5′-CGC GAA TTC GCG-3′), ds26: d(5′-CAA TCG GAT CGA ATT CGA TCC GAT TG-3′), GA triplex : d(5′-GAA AGA GAG GAG GCC TTT TTG GAG GAG AGA AAG-3′) + d(5′-CCT CCT CTC TTT C-3′), TC triplex: d(5′-CCT CCT CTC TTT CCC TTT TTC TTT CTC TCC TCC-3′) + d(5′-GAA AGA GAG GAG G-3′), TG4T: d(5′-TGG GGT-3′), TBA: d(5′-GGT TGG TGT GGT TGG-3′), HT24: d(5′-TAG GGT TAG GGT TAG GGT TAG GGT-3′), 24bcl: d(5′-CGG GCG CGG GAG GAA GGG GGC GGG-3′) and cmyc: d(5′-GGG GAG GGT GGG GAG GGT GGG GAA GGT GGG G-3′). The resulting oligonucleotides were purified by HPLC and desalted in a Sephadex (NAP-10) G25 column. Acridine-9-carboxylic acid (**1**) was obtained from Aldrich and 5-methylacridine-4-carboxylic acid (**2**) (Figure 1) was prepared following the strategy described previously [40]. Threoninol derivative (**5**) (Figure 2) was prepared following the literature [23]. NMR spectra were recorded on a Varian Mercury 400 (small compounds) or a Bruker AV-600 (oligo-nucleotides) spectrometers.

### 3.2. Solid-Phase Synthesis of Peptide Derivatives **3** and **4**

Peptide derivatives **3**, **4** were synthesized using Fmoc solid phase peptide synthesis. Fmoc-protected amino acids were obtained from Nova Biochem. The side chain of lysine was protected with the *t*-butoxycarbonyl (Boc) group and the arginine side chain was protected with the 2,2,4,6,7-pentamethyldihydrobenzofuran-5-sulfonyl (Pbf) group. Amino acids were assembled on 4-(2',4'-dimethoxyphenyl-Fmoc-aminomethyl)-phenoxy resin (Rink amide resin) solid support which allowed the cleavage in a single step treatment with 95% TFA, providing peptide amides in high yields and purities. Synthesis of amino acids derivatives: a cycle for each amino acid addition consisted of the following steps: (1) 20% piperidine in DMF; (2) 5% DIPEA in DCM; and (3) Fmoc-protected amino acid coupling with TBTU, HOBT and DIPEA catalysis then (1) 20% piperidine in DMF; (2) either compound **1** or compound **2** coupling with PyBOP, HOBT and DIPEA catalyst; and (3) cleavage with 95% TFA. The compounds were analyzed by MALDI-TOF: **3** [M^+^] = 493.3 (expected 493.3) and **4** [M^+^] = 549.3 (expected 549.3), and by HPLC obtaining a single peak of retention time 7.9 and 8.2 min, respectively. Analytical HPLC was performed using an XBridge OST C18 (Waters) column (2.5 µM, 4.6 × 5.5 mm) using a 10-min linear gradient from 9% to 45% B, and a flow rate of 1 mL/min; solution A was 5% ACN in 0.1 M aqueous TEAA, and B 70% ACN in 0.1 M aqueous TEAA. MALDI-TOF spectra were obtained using a *Perseptive Voyager DETMRP* mass spectrometer, equipped with a nitrogen laser at 337 nm using a 3 ns pulse. The matrix used contained 2,5-dihydroxybenzoic acid (DHB, 10 mg/mL in water).

### 3.3. N-((2S,3S)-1,3-Dihydroxybutan-2-yl)-5-methylacridine-4-carboxamide (**6**)

5-Methylacridine-4-carboxylic acid (**2**, 100 mg, 0.42 mmol) was reacted with EDCI (121 mg, 0.63 mmol), HOBt (85 mg, 0.63 mmol), L-threoninol (44 mg, 0.42 mmol) and 100 µL DIEA in DMF. Purification by chromatography on silica gel (10% methanol over AcOEt) yielded the compound **6** (50 mg, 35%) as a foam. UV (*λ_max_*): 249, 343, 359 and 385 nm. Fluorescence spectra: exc: 385 nm, em: 433 nm. HPLC: a single peak of retention time 9.0 min. ^1^H-NMR, δ_H_ (400 MHz, CDCl_3_): 8.99 (dd, 1H, *J* = 7.1 and 1.6 Hz), 8.87 (s, 1H), 8.15 (dd, 1H, *J* = 8.4 and 1.1 Hz), 7.90 (d, 1H, *J* = 8.4 Hz), 7.65 (dd, 1H, *J *= 8.4 and 7.1 Hz), 7.50 (dd, 1H, *J* = 8. 4 and 7.0 Hz), 4.39–4.35 (m, 1H), 4.34–4.29 (m, 1H), 4.16–4.08 (m, 2H), 2.96 (s, 3H), 1.35 (d, 3H, *J* = 6.4). [M^+^] = 324.8 (expected for C_42_H_48_N_5_O_12_P_2_ 324.4).

### 3.4. Solid-Phase Synthesis of Acridine Oligomers **7** and **8**

The assembly of L-threoninol derivatives (**7**, **8**) was carried out on Rink amide polystyrene solid support. An optimized oligonucleotide synthesis was used to build the main chain and then the intercalating agent was introduced (Scheme 2). Oligomer synthesis: a cycle for each L-threoninol backbone addition consisted of the following steps: (1) 3% trichloroacetic acid/dichlorometane; (2) coupling of compound **11** with tetrazole activation, 2 times; and (3) oxidation with 70% aq. *tert*-butyl hydroperoxide/acetonitrile (14:84 v/v) then (1) 20% piperidine in DMF; (2) either compound **1** or compound **2** coupling with PyBOP, HOBT and DIPEA catalyst; (3) 3% trichloroacetic acid/dichlorometane; and (4) cleavage with 95% TFA. The compounds were analyzed by MALDI-TOF: **7** [M^+^] = 876.8 (expected for C_42_H_48_N_5_O_12_P_2_ 876.8), **8** [M^+^] = 905.4 (expected for C_44_H_52_N_5_O_12_P_2_ 904.9) and by HPLC obtaining a peak of retention time 4.1 and 13.2 min, respectively.

### 3.5. (9H-Fluoren-9-yl)methyl (2R,3R)-1-(bis(4-methoxyphenyl)(phenyl)methoxy)-3-hydroxybutan-2-ylcarbamate (**10**)

*N*-[(9*H*-Fluoren-9-yl)methyloxycarbonyl]-L-threoninol (compound **9**, 500 mg, 1.53 mmol) was dissolved in anhydrous pyridine (10 mL) and reacted with 4,4′-*O*-dimethoxytriphenylmethyl chloride (1.84 mmol) and 4-dimethylaminopyridine (0.20 mmol, DMAP). The mixture was stirred at room temperature overnight. The reaction was quenched with methanol (0.5 mL) and the solvents were removed under reduced pressure. The residue was dissolved in dichloromethane (100 mL) and the organic phase was washed with saturated aqueous NaCl (50 mL). The solvent was evaporated and the residue was purified by chromatography on neutral aluminum oxide. The product was eluted with dichloromethane and 1% of methanol. The pure compound was obtained as an oil (733 mg, 76%). ^1^H-NMR, δ_H_ (400 MHz, CDCl_3_): 8.55–6.77 (m, 21H), 4.45–4.33 (m, 3H), 4.22 (t, 1H, *J* = 7.0 Hz), 4.08 (m, 1H), 3.76 (s, 6H), 3.43 (dd, 1H, *J* = 9.8 and 5.0), 3.25 (dd, 1H, *J* = 9.8 and 3.8 Hz), 1.16 (d, 3H, *J* = 6.3 Hz). ^13^C-NMR, δ_C_ (100 MHz, CDCl_3_): 158.6; 158.5; 1475; 145.0; 144.1; 144.0; 139.6; 130.1; 129.2; 128.3; 127.9; 127.7; 127.1; 125.2; 119.9; 113.2; 69.2; 68.9; 66.7; 58.4; 20.2; 19.5.

### 3.6. (9H-Fluoren-9-yl)methyl (4R,5R)-7-(2-cyanoethoxy)-8-isopropyl-1,1-bis(4-methoxyphenyl)-5,9-dimethyl-1-phenyl-2,6-dioxa-8-aza-7-phosphadecan-4-ylcarbamate (**11**)

Compound **10** (1.1 mmol) was dried by evaporation with anhydrous ACN. The residue was dissolved in anhydrous DCM (20 mL) and diisopropylethylamine (DIEA) (4.6 mmol) was added under argon atmosphere. The solution was cooled to 0 °C in a ice bath and 2-cyanoethoxy-*N,N*-diisopropyl-aminochlorophosphine (2.2 mmol) was added dropwise with a syringe. After the completion of the reaction, DCM (50 mL) was added and the organic layer was washed with saturated aqueous NaCl (100 mL). The solvent was evaporated under reduced pressure and the residue was purified by column chromatography on neutral aluminum oxide. The product was eluted with hexane/ethyl acetate 2:3. The desired compound was obtained (556 mg, 61%). ^1^H-NMR, δ_H_ (400 MHz, CDCl_3_): 7.77–6.80 (m, 21H), 5.09 and 4.99 (d, 1H, *J* = 9.6 and 9.5 Hz, respectively, two isomers), 4.41–4.38 (m, 3H), 4.36–4.29 (m, 2H), 4.22 (t, 1H, *J* = 7.0 Hz), 3.93–3.79 (m, 1H), 3.79 (s, 6H), 3.55–3.34 (m, 4H), 2.36 (t, 2H, *J* = 6.3 Hz), 1.27–1.11 (m, 15H). ^31^P-NMR, δ_P_ (81 MHz, CDCl_3_): 148.88, 148.63 (phosphoric acid as external reference). ^13^C NMR, δ_C_ (100 MHz, CDCl_3_): 158.6; 158.4; 144.8; 144.9; 144.1; 144.0; 136.0; 130.1; 130.0; 129.1; 128.3; 127.9; 127.7; 127.1; 127.0; 126.8; 125.1; 119.9; 117.6; 113.2; 69.0; 68.9; 66.6; 58.4; 56.2; 55.2;43.2; 43.1; 24.7; 24.6; 24.4; 24.3; 20.3, 19.5; 19.4.

### 3.7. Competitive Dialysis Assays

Slide-A-Lyzer Mini Dialysis Units 3500MWCO were purchased from Pierce. A total 200 mL of the dialysate solution containing 1 μM of the compound was used for each competition dialysis assay. A volume of 100 μL of 50 μM monomeric unit of each of oligonucleotide sequence was placed in the dialysis unit. Potassium phosphate buffer containing 185 mM NaCl, 185 mM KCl, 2 mM NaH_2_PO_4_, 1 mM Na_2_EDTA and 6 mM Na_2_HPO_4_ at pH 7 was used for all experiments.

The samples were allowed to equilibrate with continuous stirring at room temperature overnight. Dialysis samples were transferred to an Eppendorf tube. In order to measure the compound entered in the dialysis unit, dialysis samples were treated with a snake venom phosphodiesterase to release the intercalating compound as described previously [25].

Finally, the fluorescence of each sample was measured (*λ_ex_* and *λ_em_* were set to 252 and 435 nm for compounds **1**, **5** and **7** and to 258 and 460 nm for compounds **2**, **3**, **4**, **6** and **8** and normalized for each compound.

### 3.8. Cell Viability Assays

The *in vitro* cytotoxicity of the compounds (**1**, **2**, **3**, **4**, **5**, **6**, **7** and **8**) was evaluated by colorimetric assays with a tetrazole salt (MTT) on the HTB-38 (human colon carcinoma) cell line. The cell line was cultured in RPMI and supplemented with 10% fetal calf serum and 200 mM L-glutamine. Cells were grown in a humidified atmosphere of air containing 5% CO_2_ at 37°. Cells were plated in triplicate wells (1.5 × 10^4^ cells per well) in 100 µL of growth medium in 96-well plates and allowed to proliferate for 24 h. They were then treated with increasing concentrations of the compounds. After 72 h incubation, 20 μM MTT (5 mg/mL in phosphate buffer saline 10%) was added for additional 4 h. Absorbance at 570 nm was measured on a multiwell plate reader after removing the medium and addition of 50 μL of dimethyl sulfoxide. Cell viability was expressed as a percentage of control and IC_50_ was determined as the concentration of the drug that produced 50% reduction of absorbance at 570 nm.

### 3.9. NMR Spectroscopy

The NMR spectra of oligonucleotides were recorded at 25 °C on a Bruker AV-600 spectrometer equipped with a *z*-gradient triple resonance TXI and were processed with TOPSPIN v.1. The instrument was operated at a frequency of 600.10 MHz for ^1^H. ^1^H chemical shifts (δ_H_) were measured in ppm and referenced to external DSS (sodium 2,2-dimethyl-2-silapentane-5-sulfonate salt) set for 0.00 ppm. Estimated accuracy for protons is within 0.02 ppm. The samples for NMR measurements were dissolved in 500 μL H_2_O/D_2_O (9:1) containing 25 mM KH_2_PO_4_, 150 mM KCl and 1 mM EDTA (pH 6.7) for the quadruplex d(5′-TTAGGG-3′)_4_ and containing 10 mM KH_2_PO_4_, 70 mM KCl and 0.2 mM EDTA (pH 7.0) for the double helix d(5′-CGATCG-3′)_2_. The final concentration of the oligonucleotide solutions ranged between 0.7 and 0.6 mM. A stock solution of **2** and **8** was prepared in DMSO-d_6_ at the concentrations of 10 and 20 mM, respectively. ^1^H-NMR titration was performed by adding increasing amounts of **2** and **8** to the oligonucleotide solutions at an R= [Ligand]/[DNA] ratio equal to 0, 0.25, 0.5, 0.75, 1, 2 and 3.

## 4. Conclusions

Several compounds with the 5-methylacridine-4-carboxamide core group present in a DNA intercalating dual topoisomerase I/II inhibitor (DACA) derivative were prepared and their anti-proliferative properties were studied. Solid-phase methods were used for the rapid generation of the 5-methylacridine-4-carboxamide derivatives. As the protonable dimethylamino group was considered relevant for the biological activity [22], we first studied the replacement of this group by natural amino acids with protonable groups like lysine and arginine. Unfortunately, the resulting peptide-acridine compounds lost DNA binding capacity and thus were inactive. In contrast, the threoninol derivative carrying the 5-methylacridine-4-carboxamide unit retained the anti-proliferative properties of the 5-methylacridine-4-carboxylic acid. Linking two units of the 5-methylacridine-4-carboxamide with a threoninol phosphate backbone generated the most active compound, in spite of the presence of the negative phosphate backbone. This finding demostrates that linking several intercalating units with a negative backbone may indeed be a useful strategy to obtain novel DNA-intercalating drugs as the DNA-binding properties are not negatively affected and compounds have increased solubility in aqueous solutions.
